# Mean Platelet Volume/Lymphocyte Ratio as a Prognostic Indicator for HBV-Related Decompensated Cirrhosis

**DOI:** 10.1155/2020/4107219

**Published:** 2020-07-02

**Authors:** JianPing Wu, WeiLin Mao, XinKe Li

**Affiliations:** ^1^Department of Clinical Laboratory, The First Affiliated Hospital, College of Medicine, Zhejiang University, Hangzhou, Zhejiang 310003, China; ^2^Department of Radiation Oncology, The First Affiliated Hospital, College of Medicine, Zhejiang University, Hangzhou, 310003 Zhejiang, China

## Abstract

**Aim:**

To evaluate the prognostic role of the mean platelet volume/lymphocyte ratio (MPVLR) for mortality in patients with hepatitis B virus-related decompensated cirrhosis (HBV-DeCi).

**Methods:**

The medical records of 101 patients with HBV-DeCi were retrospectively reviewed, and their baseline clinical and laboratory characteristics were extracted. The predictive value of the MPVLR for death was estimated using receiver operating characteristic curve analysis and a multivariate logistic regression model.

**Results:**

Patients with HBV-DeCi in the high-MPVLR group exhibited significantly increased 90-day mortality compared with that of the patients within the low-MPVLR group, and MPVLR was an independent predictor of 90-day mortality in patients with HBV-DeCi.

**Conclusions:**

Increased MPVLR is associated with poor outcomes in patients with HBV-DeCi and might be a useful component of future prognostic scores.

## 1. Introduction

Hepatitis B virus (HBV) infection is the main cause of liver fibrosis and cirrhosis. In China, the incidence of decompensated cirrhosis is approximately 3% per year [1]. HBV-related decompensated cirrhosis (HBV-DeCi) is accompanied by various complications, which are responsible for death [2]. Liver transplantation is the most effective therapy for patients with disease that has progressed to DeCi. However, the shortage of donor organs and the high medical expense have limited its clinical application. Therefore, finding a readily available and simple marker for HBV-DeCi prognosis and disease monitoring is urgently needed.

Mean platelet volume (MPV) is routinely measured by CBC analyzers and a parameter reflecting platelet size. MPV is also a marker of platelet function and activation [3, 4]. MPV has been found to be highly associated with poor outcomes in different clinical settings [5–9]. Moreover, several studies have reported that MPV predicts the severity, fibrosis, and inflammation in chronic viral hepatitis, especially in cases of HBV [10]. For example, an elevated MPV is associated with worse outcomes in patients with HBV-associated liver failure or HBV-DeCi [11, 12]. Additionally, according to previous research, a low lymphocyte count is associated with patient frailty, leading to poor outcome. Some studies have demonstrated that low lymphocyte count is associated with prognosis in several cancers, such as hematological malignancy, breast cancer, and renal cell cancer [13–15], and it was reported that the pretransplant lymphocyte count is one of the prognostic factors in liver transplant recipients [16, 17]. Moreover, Ceccato et al. found that a low lymphocyte count can be defined as an independent predictive factor for mortality in patients with ICU-acquired pneumonia [18]. Recently, the MPV/lymphocyte ratio (MPVLR) was considered an objective and simple biomarker of inflammation and was initially proposed by Hudzik et al. [19] in 2016. They reported that MPVLR plays an important role in developing intravascular thrombi in ST elevation myocardial infarction. In addition, recent studies have demonstrated that the MPVLR level has clinical implications in various pathologies, such as systemic inflammation, myocardial infarction, and impaired coronary collateral circulation [20, 21]. Previous studies have reported that systemic inflammation occurs frequently in patients with advanced liver cirrhosis and may be associated with worse outcomes [22]. Inflammation-based prognostic factors, for example, C-reactive protein [23], interleukin-6 [24], and systemic inflammatory response syndrome [25], have been proven to be beneficial in cirrhosis mortality prediction. Based on these findings, we proposed that MPVLR as a novel marker of inflammation may correlate with prognosis in patients with HBV-DeCi. Therefore, we planned to investigate the MPVLR in patients with HBV-DeCi and evaluate its prognostic value in these patients.

## 2. Materials and Methods

### 2.1. Patients

This retrospective study involved 151 newly diagnosed patients with HBV-DeCi admitted to our hospital from January 2018 to May 2019. Liver decompensation was defined as the rapid development of one or more major complications (i.e., large ascites, hepatic encephalopathy (HE), hepatorenal syndrome (HRS), and variceal bleeding) of liver disease [26]. Of these patients, 50 were excluded because they had alcoholic liver disease (*n* = 15), autoimmune hepatitis (*n* = 3), drug-induced liver injury (*n* = 4), other viral infections (hepatitis A or C or HIV infection) (*n* = 7), malignancy (*n* = 2), drug use history (aspirin, clopidogrel, or steroids) (*n* = 3), cardiac disease (*n* = 4), heart failure (*n* = 2), hematological disease (*n* = 2) or had received antiviral or immunomodulatory therapy in the past 3 months (*n* = 8). Eventually, 101 patients were enrolled in this investigation. The primary outcome was 90-day mortality.

The study was performed according to the Declaration of Helsinki; the procedures were approved by the Ethics Committee of the First Affiliated Hospital of Zhejiang University College of Medicine.

### 2.2. Clinical Data Extraction

Retrospectively collected data included patient demographics and clinical data (i.e., age, sex, and complications related to liver disease, such as ascites, HE, and HRS) and laboratory variables. Laboratory parameters, including total protein, albumin, alanine aminotransferase (ALT), aspartate aminotransferase (AST), total bilirubin, creatinine and the international normalized ratio (INR), MPV, lymphocyte count, and PLT counts (PLTs), were obtained from medical records. MPVLR was calculated as MPV divided by lymphocyte count. Additionally, hepatic function was evaluated using the model for end-stage liver disease (MELD) score and the Child-Pugh score (CP), which were calculated as previously described [27, 28].

### 2.3. Statistical Analysis

Quantitative data are reported as the means ± SD or medians (interquartile range (IQR)). Qualitative data are expressed as percentages. The differences between patients with HBV-DeCi were assessed with an independent sample *t*-test, the Mann–Whitney *U*-test or the *χ*^2^ test, as appropriate. The prediction of 90-day mortality by different variables was evaluated using area under the receiver operating characteristic (AUROC) curves. To identify potential correlates of mortality, univariate analyses were first performed in demographic variables as well as clinical variables. A multivariate linear regression was subsequently performed to include variables with *P* < 0.10 in the univariate analysis. Finally, a stepwise selection with the same set of variables was conducted using *P* < 0.05 as a criterion for inclusion. SPSS 18 (Chicago, IL, USA) and MedCalc 11.6.1 (Ostend, Belgium) were used to analyze the data. Statistical significance was set at *P* < 0.05.

## 3. Results

### 3.1. Baseline Characteristics

Among the 101 patients with HBV-DeCi (mean age 52.9 ± 11.7 years, 17.8% women), the most common complication was ascites (*n* = 69, 68.3%), followed by gastrointestinal bleeding (*n* = 25, 24.8%), HRS (*n* = 10, 10.0%), bacterial infections (*n* = 25, 24.8%), and HE (*n* = 1, 1.0%). The demographic, clinical, and laboratory characteristics of the participants are presented in Table 1. The MPVLR ranged from 5.2 to 84.0 (median: 15.1).

### 3.2. Characteristics and Baseline Factors Related to MPVLR Levels

Patients with HBV-DeCi were divided into two groups based on the median MPVLR: the low group (MPVLR ≤ 15.1) and the high group (MPVLR > 15.1). The clinical and laboratory characteristics of the two groups are shown in Table 2. The MELD score in the high-MPVLR group was higher than that in the low-MPVLR group. Moreover, patients with higher MPVLR had lower PLTs and had higher INR and 3-month mortality compared to those of the low-MPVLR group. No significant differences in age, sex, total protein, albumin, creatinine, total bilirubin, or AST were detected.

### 3.3. Comparison of MPVLR between Nonsurviving and Surviving Patients and the Evaluation of MPVLR as Prognostic Factor

Of all 101 patients, 63 survived and 38 died, with a mortality of 37.6%. The cause of death was hepatic failure in ten patients, upper gastrointestinal bleeding in fifteen, HE in one, HRS in three, and infection in nine. The comparison of demographic, clinical, and laboratory parameters between nonsurvivors and survivors is described in Table 3. Patients who died had a higher MPVLR than surviving patients did (median 22.1, interquartile range (IQR) 15.8–37.8 vs. 11.5, 8.8–18.6, *P* < 0.001). Moreover, a higher MPV level (median 17.6, IQR 15.0–19.7 vs. 15.1, 13.0–17.7, *P* = 0.008) and a lower lymphocyte count (median 0.75, IQR 0.50–1.10 vs. 1.30, 0.73–1.88, *P* < 0.001) were observed in the nonsurviving group compared with the surviving group. These data suggested that the higher MPVLR in the nonsurviving group could be attributed to the increased MPV level and decreased lymphocyte count. In addition, nonsurvivors also had a higher total bilirubin (*P* = 0.001), CP score (*P* = 0.014), INR (*P* = 0.036), and MELD (*P* < 0.001) and a lower albumin (*P* = 0.044) and PLTs (*P* = 0.002) than those of the surviving patients. No significant differences between the two groups in terms of age, sex, total protein, creatinine, AST, or ALT were detected (all *P* > 0.05).

**Figure 1:**
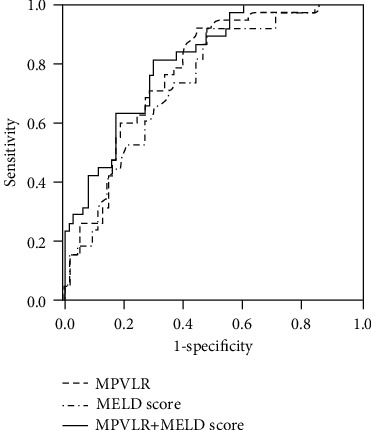
Receiver operating characteristic curves indicating the relative efficiencies of MELD score, MPVLR, and the combination of both for predicting 3-month mortality in patients with HBV-DeCi.

To identify the prognostic factors, univariate and multivariate analyses were carried out. For univariate analysis, a higher MELD (odds ratio (OR) = 1.158, 95% confidence interval (95% CI) 1.071–1.252; *P* < 0.001), MPVLR (OR = 1.076, 95% CI 1.035–1.119; *P* < 0.001), and CP score (OR = 1.309, 95% CI 1.042–1.645; *P* = 0.021) and a lower lymphocyte count (OR = 0.231, 95% CI 0.096–0.557; *P* = 0.001) and PLTs (OR = 0.988, 95% CI 0.980–0.997; *P* = 0.007) were associated with the mortality of HBV-DeCi patients. In the multivariate analysis, only the MELD (OR = 1.169, 95% CI 1.068–1.279; *P* = 0.001) and the MPVLR (OR = 1.077, 95% CI 1.032–1.124; *P* = 0.001) were independent predictors of mortality (Table 4). ROC curve analysis was performed to evaluate the relative efficiencies of the MPVLR and MELD score for predicting mortality (Figure 1). The MELD score had the best cut-off value of 17.2, with a sensitivity of 50.0% and a specificity of 81.0%. MPVLR had the best cut-off value of 19.4 with a sensitivity of 60.5% and a specificity of 81.0%. The predictive powers of the MPVLR and MELD score for mortality were not significantly different, which is reflected by the similar AUC values (0.779 (95% CI 0.685–0.855) for MPVLR vs. 0.740 (95% CI 0.643–0.822) for MELD score; *P* = 0.485). When MPVLR and MELD score were analyzed in combination, the AUC was 0.807—slightly higher than that of MPVLR (*Z* = 0.745, *P* = 0.456) and MELD score (*Z* = 1.643, *P* = 0.100)—and the specificity (82.5%) and the sensitivity (63.2%) are not significantly improved.

## 4. Discussion

We first investigated the value of MPVLR in patients with HBV-DeCi, and the results indicated that elevated MPVLR was associated with increased mortality. Furthermore, MPVLR was an independent predictor of mortality in these patients.

The CP and MELD scoring systems have been used widely to predict the outcomes of patients with liver disease; however, they both have some limitations [24, 29]. Two variables (i.e., ascites and HE) included in the CP score are subjective, which may reduce the accuracy of the assessment [29]. In our study, the CP score was not an independent predictor of 3-month mortality in the multivariate analysis. This is in accordance with Fontana et al., who demonstrated that the CP score did not predict early mortality in patients with HBV-DeCi [30]. Indeed, in patients with liver failure, the CP score showed the lowest discriminative ability in predicting mortality among all of the scores evaluated, including the MELD, APACHE-II, and SOFA scores [31]. The MELD score incorporates 3 laboratory variables, namely, total bilirubin, INR, and creatinine, and it is used to assign priorities to cadaveric organs on the transplant waiting list and is the prognostic tool for assessing the 3- to 6-month survival of patients with liver failure [32]. However, approximately 15% to 20% of candidates for liver transplantation are not well served by the MELD score. This is because important factors (i.e., HE, HRS, or variceal bleeding) that can affect the prognosis of patients are not taken into consideration in the MELD score [24]. In this study, the MELD score was an independent predictor of 3-month mortality in the multivariate analysis. Our study showed that the nonsurviving patients had a higher MPVLR than the surviving patients. Furthermore, multivariate logistic regression analyses showed that the MPVLR was a novel predictor of 3-month outcomes, and the prediction power of the MPVLR was slightly higher than that of the MELD. By comparison, the MPVLR, in which only blood samples need to be tested, is more convenient and inexpensive than the CP and MELD scores. Previous studies have reported that age [33], plasma D-dimer level [34], serum cystatin C level [35], and total bilirubin level [36] are associated with poor outcomes in cirrhosis patients. The present study complements these studies, adding high MPVLR as a predictor of prognosis in patients with HBV-DeCi.

Several mechanisms were raised to explain the correlation between elevated MPVLR levels and worse outcomes in patients with HBV-DeCi. First, our results showed that MPV was significantly increased in nonsurviving patients compared with surviving patients. MPV is not only an index of platelet function and activity but also a new marker of inflammation [37, 38]. Several researchers have investigated the value of MPV in HBV-related liver diseases [10]. For example, Han et al. [11] indicated that the MPV is superior to the MELD in predicting the mortality rate in patients with HBV-associated liver failure. Next, Ma et al. [12] demonstrated that MPV is a predictor of 3-month mortality independent of the CP and MELD score in patients with HBV-DeCi. Previous studies have reported that inflammatory responses are correlated with liver disease development and may be associated with poor outcomes. Researchers have therefore suggested that an elevation in MPV is due to the consumption of large active platelets during inflammation in liver diseases [37, 39, 40]. Suvak et al. showed that MPV is an effective index of systemic inflammation in patients with cirrhosis with ascitic fluid infection [37]. We found 69 patients with ascites, of whom 25 had bacterial infections in our cohort. However, in the present study, multivariate analysis showed that the MPV did not successfully predict survival outcomes. Second, our results also indicated that a lower lymphocyte count was found in the nonsurviving group than in the surviving group. Previous research reported that a low lymphocyte count may be related to poor nutritional status and impaired immune response in patients with liver disease [41]. Moreover, Leithead and colleagues showed that a lower lymphocyte count was a predictor of waitlist death for liver transplantation [42]. Similar to the MPV, the lymphocyte count was also not considered a prognostic factor for mortality in the multivariate analysis in the present study. We think this result is mainly due to the MPVLR being a ratio; it is more stable than individual variables, which may be affected by several factors, such as dehydration or overhydration. Our results suggest that MPVLR had more accuracy than MPV or lymphocyte count alone considering its prognostic ability in patients with HBV-DeCi. In the current study, the association of higher MPVLR with parameters that reflect the severity of liver disease indicates higher MPVLR to be predictive of the severity and progression of liver injury among patients with HBV-DeCi. The current results demonstrated that the higher MPVLR was predominantly due to increased MPV and a decreased lymphocyte count. Therefore, we proposed that an aberrant MPVLR might reflect systemic inflammation and the severity of immune injury and may influence the prognosis of patients with HBV-DeCi.

However, our study has potential limitations. First, because this was a retrospective study from a single center, potential selection bias might exist. Second, the analysis was not conducted on the training cohort or the validation cohort separately. Thus, further prospective studies need to be performed in multiple centers to verify the predictive value of the MPVLR in patients with HBV-DeCi. Finally, we did not evaluate some inflammatory markers, such as C-reactive protein and interleukin-6. The evaluation of these markers helped to elucidate the mechanism underlying the findings presented here. Despite these limitations, we present the first study that focused on the predictive value of MPVLR in the setting of HBV-DeCi.

In summary, the current study indicated that the MPVLR was a simple and objective biomarker that could predict the short-term mortality rate of patients with HBV-DeCi. MPVLR may provide valuable information to supplement conventional approaches of assessing the disease condition in these patients. It represents a useful tool in clinical practice, to assess patient prognosis and help clinicians identify individuals in need of greater care. Future studies are warranted to validate the current findings.

## Figures and Tables

**Table 1:** Clinical characteristics of studied subjects.

	Total patients (*n* = 101)
Gender (male/female)	83/18
Age (years)	52.9 ± 11.7
Total protein (g/L)	60.4 ± 7.7
Albumin (g/L)	29.6 ± 5.2
ALT (U/L)	32.0 (18.0–49.5)
AST (U/L)	49.0 (31.5–73.5)
Serum creatinine (*μ*mol/L)	74.0 (61.0–89.0)
Total bilirubin (*μ*mol/L)	50.0 (22.5–131.0)
INR	1.51 ± 0.39
MPV (fl)	16.0 (13.6–18.9)
Lymphocyte count (×10^9^/L)	1.00 (0.65–1.40)
MPVLR	15.1 (9.9–25.7)
PLTs (×10^9^/L)	76.0 (38.0–123.0)
MELD score	13.5 (8.6–17.8)
CP score	9.0 (8.0–11.0)
Modes of decompensation	
HE, *n* (%)	1 (1.0%)
HRS, *n* (%)	10 (10.0%)
Ascites, *n* (%)	69 (68.3%)
Variceal bleeding, *n* (%)	25 (24.8%)
3-month mortality, *n* (%)	38 (37.6%)

Data are expressed as *n*, mean ± SD, or median (interquartile range). Abbreviations: ALT: alanine aminotransferase; AST: aspartate aminotransferase; INR: international normalized ratio; MPV: mean platelet volume; MPVLR: mean platelet volume/lymphocyte ratio; PLTs: platelet counts; MELD score: model for end-stage liver disease score; CP score: Child-Pugh score; HE: hepatic encephalopathy; HRS: hepatorenal syndrome; HBV-DC: HBV-related-decompensated cirrhosis.

**Table 2:** Clinical and laboratory characteristics of patients with various MPVLR values on admission.

	Low group (MPVLR ≤ 15.1, *n* = 50)	High group (MPVLR > 15.1, *n* = 51)	*P*
Gender (male/female)	9/41	9/42	0.831
Age (years)	51.4 ± 13.0	54.4 ± 10.3	0.193
Total protein (g/L)	60.0 (55.3-66.6)	59.7 (55.8-64.5)	0.847
Albumin (g/L)	30.0 (25.8-33.2)	28.9 (26.4-32.1)	0.407
AST (U/L)	52.5 (35.0-74.0)	47.0 (29.0-70.8)	0.262
Serum creatinine (*μ*mol/L)	74.0 (62.0-84.0)	74.0 (60.3-98.5)	0.755
Total bilirubin (*μ*mol/L)	41.5 (21.0-103.0)	68.0 (27.8-154.3)	0.097
INR	1.44 ± 0.36	1.60 ± 0.41	0.032
MPV (fl)	15.1 (13.8-17.5)	16.8 (13.6-19.5)	0.039
Lymphocyte count (×10^9^/L)	1.40 (1.20-2.10)	0.70 (0.50-0.80)	<0.001
MPVLR	9.9 (8.4-12.0)	25.3 (18.8-37.6)	<0.001
PLTs (×10^9^/L)	91.0 (65.0-159.0)	44.0 (28.8-80.0)	<0.001
MELD score	10.9 (6.3-17.8)	15.2 (11.8-17.8)	0.011
CP score	9.0 (8.0-11.0)	9.0 (8.3-11.0)	0.140

Data are expressed as *n*, mean ± SD, or median (interquartile range). Abbreviations: ALT: alanine aminotransferase; AST: aspartate aminotransferase; INR: international normalized ratio; MPV: mean platelet volume; MPVLR: mean platelet volume/lymphocyte ratio; PLTs: platelet counts; MELD score: model for end-stage liver disease score; CP score: Child-Pugh score; HBV-DC: HBV-related-decompensated cirrhosis.

**Table 3:** Comparison of the surviving and nonsurviving patients with HBV-DeCi.

	Nonsurviving patients (*n* = 38)	Surviving patients (*n* = 63)	*P*
Gender (female/male)	7/31	11/52	0.884
Age (years)	53.9 ± 10.4	52.3 ± 12.5	0.453
Total protein (g/L)	59.6 (54.4-63.7)	59.9 (56.0-66.4)	0.497
Albumin (g/L)	28.9 (24.7-31.2)	30.1 (26.4-33.9)	0.044
ALT (U/L)	32.5 (17.0-60.0)	32.0 (20.0-48.0)	0.715
AST (U/L)	52.5 (34.0-86.0)	48.0 (29.5-72.8)	0.348
Serum creatinine (*μ*mol/L)	74.5 (66.0-100.0)	73.0 (58.3-83.0)	0.147
Total bilirubin (*μ*mol/L)	97.5 (50.0-203.0)	36.0 (21.0-93.0)	0.001
INR	1.62 ± 0.38	1.46 ± 0.39	0.036
MPV (fl)	17.6 (15.0-19.7)	15.1 (13.0-17.7)	0.008
Lymphocyte count (×10^9^/L)	0.75 (0.50-1.10)	1.30 (0.73-1.88)	<0.001
MPVLR	22.1 (15.8-37.8)	11.5 (8.8-18.6)	<0.001
PLTs (×10^9^/L)	47.0 (31.0-80.0)	83.0 (54.3-151.0)	0.002
MELD score	17.3 (12.2-20.5)	10.7 (6.9-16.5)	<0.001
CP score	11.0 (9.0-11.0)	9.0 (8.0-11.0)	0.014

Data are expressed as *n*, mean ± SD, or median (interquartile range). Abbreviations: ALT: alanine aminotransferase; AST: aspartate aminotransferase; INR: international normalized ratio; MPV: mean platelet volume; MPVLR: mean platelet volume/lymphocyte ratio; PLTs: platelet counts; MELD score: model for end-stage liver disease score; CP score: Child-Pugh score; HBV-DC: HBV-related-decompensated cirrhosis.

**Table 4:** Multivariate analysis to identify the independent factors associated with outcomes in patients with HBV-DeCi.

	Univariable	95% CI	*P*	Multivariable	95% CI	*P*
	Odds ratio			Odds ratio		
Age (years)	1.014	0.979-1.050	0.441			
CP score	1.309	1.042-1.645	0.021			
MELD score	1.158	1.071-1.252	<0.001	1.169	1.068-1.279	0.001
MPV (fl)	1.153	1.030-1.290	0.014			
Lymphocyte count (×10^9^/L)	0.231	0.096-0.557	0.001			
MPVLR	1.076	1.035-1.119	<0.001	1.077	1.032-1.124	0.001
PLTs (×10^9^/L)	0.988	0.980-0.997	0.007			

Abbreviations: CP score: Child-Pugh score; MELD score: model for end-stage liver disease score; MPV: mean platelet volume; MPVLR: mean platelet volume/lymphocyte ratio; PLTs: platelet counts; CI: confidence interval; HBV-DC: HBV-related-decompensated cirrhosis.

## Data Availability

The data are available upon reasonable request.

## Abbreviations

ALT: Alanine aminotransferase
AST: Aspartate aminotransferase
AUCs: Areas under the curve
CP score: Child-Pugh score
DeCi: Decompensated cirrhosis
HBV: Hepatitis B virus
HE: Hepatic encephalopathy
INR: International normalized ratio
LC: Liver cirrhosis
MELD score: Model for end-stage liver disease score
MPV: Mean platelet volume
MPVLR: Mean platelet volume-to-lymphocyte ratio
PLTs: Platelet
ROC: Receiver operating characteristic.
